# Ceftriaxone as an Alternative Therapy for the Treatment of Methicillin-Susceptible *Staphylococcus aureus* Bacteremia after Initial Clearance of Bloodstream Infection

**DOI:** 10.1155/2021/8884685

**Published:** 2021-04-26

**Authors:** Katie E. Barber, Rachel A. Cramer, Allison M. Bell, Jamie L. Wagner, Kayla R Stover

**Affiliations:** ^1^University of Mississippi School of Pharmacy, Department of Pharmacy Practice, 2500 North State Street, Jackson, MS 39216, USA; ^2^University of Mississippi Medical Center, Department of Medicine- Infectious Diseases, 2500 North State Street, Jackson, MS 39216, USA

## Abstract

**Introduction:**

*Staphylococcus* spp. represent the leading cause of hospital-acquired infections and second-most frequent pathogen in bloodstream infections. Methicillin-susceptible *S. aureus* (MSSA) comprise approximately half of all *S. aureus* isolates. Standard-of-care therapies (SOCTs) display high treatment success but require frequent dosing, are problematic in penicillin allergic patients, and are nephrotoxic. Ceftriaxone may represent an alternative treatment option.

**Methods:**

Adult patients hospitalized from January 2015 through June 2016 with positive MSSA blood cultures and treated with SOCT or ceftriaxone for ≥48 hours were included. Exclusion criteria were receipt of vancomycin or concomitant systemic antimicrobials with activity against MSSA, polymicrobial infections, and pregnant patients. Additional data collected included demographics, source/site of infection, and treatment. The primary endpoint was clinical cure (normalization of white blood cell count and temperature within 7 days and clearance of bloodstream within 7 days). Readmission within 60 days, length of stay, and discharge disposition were collected.

**Results:**

A total of 43 patients were included: 23 receiving SOCT and 20 receiving ceftriaxone group. Sixteen patients received SOCT prior to ceftriaxone while 4 patients were initiated on ceftriaxone. Clinical cure was observed in 18/23 (78%) and 10/20 (50%), respectively (*P*=0.052). Clinical failure was driven by leukocytosis despite clearance of their bloodstream infection in 3/23 (13%) SOCT group compared to 8/20 (40%) in the ceftriaxone group (*P*=0.043). Six patients (SOCT: 2, ceftriaxone: 4; *p*=0.669) had infection-related readmissions, and 1 death per group was observed.

**Conclusion:**

Ceftriaxone poses a reasonable alternative to consider for MSSA bacteremia when cost and feasibility are concerns for outpatient parenteral therapy after initial clearance of bloodstream infections.

## 1. Introduction

*Staphylococcus aureus* is a virulent pathogen and a cause of great morbidity and mortality worldwide, leading to complications in 11–53% of patients [1, 2]. This aggressive microorganism often disseminates, resulting in infective endocarditis, prosthetic infections, catheter-related bloodstream infections, and bacteremia [3]. Mortality rates of methicillin-susceptible *S. aureus* (MSSA) and methicillin-resistant *S. aureus* (MRSA) bacteremia are estimated to be 25% and 34%, respectively [4].

Currently, standard-of-care therapy (SOCT) for MSSA bacteremia includes intravenous (IV) anti-staphylococcal penicillins (ASPs) for 4 weeks or longer, depending on infection site and presence of metastatic complications [5]. ASPs, predominantly nafcillin and oxacillin, require frequent dosing (every 4 hours), are costly, and are associated with a high likelihood of adverse effects [5]. These frequent dosing intervals place a high burden on nursing staff. In addition, for patients discharged home requiring IV antibiotic treatment for several weeks, a medication requiring administration every 4 hours is often not practical or feasible, when alternative dosing regimens are not available. Average wholesale prices (AWPs) of nafcillin (2 g, 6 times daily) and oxacillin (2 g, 6 times daily) are $224 and $174 per day, respectively [6, 7]. Additionally, adverse effects associated with these agents include hepatotoxicity (22%), neutropenia (17%), rash (32%), and renal dysfunction (33%) [8–10]. These adverse effects generally occur after 2 weeks of therapy, resulting in premature discontinuation in 33.8% of patients [8].

An alternative to ASP treatment for MSSA bacteremia is a cephalosporin. Cefazolin has been the most frequently studied cephalosporin in the setting of MSSA [8, 10–12]. Compared to ASPs, cefazolin has an improved dosing regimen with administration every 8 hours. Additionally, cefazolin (2 g, 3 times daily) is cheaper than ASPs with an AWP of $45 per day [13]. Patients treated with cefazolin for MSSA bacteremia experience fewer adverse effects than those treated with ASPs; premature discontinuation was observed in only 6.7% of patients [5, 8]. Most studies have shown similar efficacy between ASPs and cephalosporins. However, cefazolin is hindered by an inoculum effect and so may be less efficacious in disease states with a high bacterial burden, including infective endocarditis. A systematic review and meta-analysis demonstrated lower 30-day mortality with ASPs in an unadjusted analysis; however, propensity-adjusted score data showed no difference in 30-day mortality between ASPs and cephalosporins [14].

While cefazolin improves the dosing regimen, decreases cost, and decreases adverse effects when compared to ASPs, it still requires frequent dosing. This is a great burden for outpatient antibiotic services and on patients themselves. Ceftriaxone presents an attractive alternative to ASPs or cefazolin and can be dosed once daily. Few studies have investigated the use of ceftriaxone as an alternative to SOCT [15–17]. Additionally, ceftriaxone (2 g, daily) has a low AWP of $29 per day. Its adverse effect profile is favorable with no renal dosage adjustment requirements, and it is well tolerated [18]. In this retrospective study, we sought to evaluate the efficacy of ceftriaxone compared to SOCT in patients hospitalized with MSSA bacteremia.

## 2. Methodology

### 2.1. Study Design

All adult inpatients with positive blood cultures for MSSA were evaluated for inclusion in this single-center cohort study. The primary objective was to determine clinical cure rates for MSSA bacteremia with ceftriaxone versus SOCT. Clinical cure was a composite endpoint defined as resolution of fever, normalization of white blood cell (WBC) count, and negative blood cultures within 7 days of starting therapy similar to prior studies of *S. aureus* infections [19–21]. Secondary outcomes included microbiological cure rates, length of stay, and disease recurrence. Microbiological cure rate was defined as negative blood cultures within 7 days of starting therapy. Disease recurrence was defined as the incidence of a positive blood culture with MSSA after an initial clearance within 6 months of completion of therapy. The ceftriaxone group was defined as patients that definitively received ceftriaxone therapy.

### 2.2. Patient Selection

Patients admitted and diagnosed with MSSA bacteremia between February 1, 2015, and January 31, 2016, were eligible for inclusion. Patients were identified through TheraDoc® clinical decisions support software and were stratified into two treatment groups: SOCT or ceftriaxone/SOCT followed by ceftriaxone therapy. SOCT was defined as treatment with nafcillin, oxacillin, or cefazolin. Patients were included if they received ≥48 hours of therapy and treated with the appropriate duration for the source of their bloodstream infection. Excluded patients were those treated definitively with vancomycin for their MSSA infection, received alternative antimicrobials with *in vitro* MSSA activity, patients with polymicrobial infections, or pregnant women. Patients could only be included for their first MSSA bloodstream infection during the study period. No written consent was obtained from the patients as there are no patient identifiable data included in this case series.

### 2.3. Data Collection

Patient demographics, source of infection, infection acquisition, and treatment regimen were collected. Infection acquisition was defined as follows: community if the patient came in with the infection; nosocomial if the infection developed ≥48 hours of admission; healthcare defined as hospitalization in an acute care hospital for two or more days within 90 days of the infection, resided in a nursing home or long-term care facility, received recent intravenous antibiotic therapy, chemotherapy, or wound care within the past 30 days of the current infection, or attended a hospital or hemodialysis clinic. Additional data collected included clinical cure, microbiological cure, readmission for another MSSA infection within 60 days of clearance of the initial bacteremia, length of stay, retreatment within 3 months of discharge, mortality, and discharge disposition.

### 2.4. Statistical Analysis

All consecutive patients meeting inclusion criteria were analyzed. Descriptive statistics were calculated; continuous, normally distributed data were analyzed using the Student's *t*-test, and non-normal data were analyzed using the Mann–Whitney *U* test. Chi square or Fisher's exact test was used to compare nominal data. A 2-sided *P* value of <0.05 was considered statistically significant. Statistical analysis was performed using SPSS software version 24.0 (IBM).

## 3. Results

**Figure 1:**
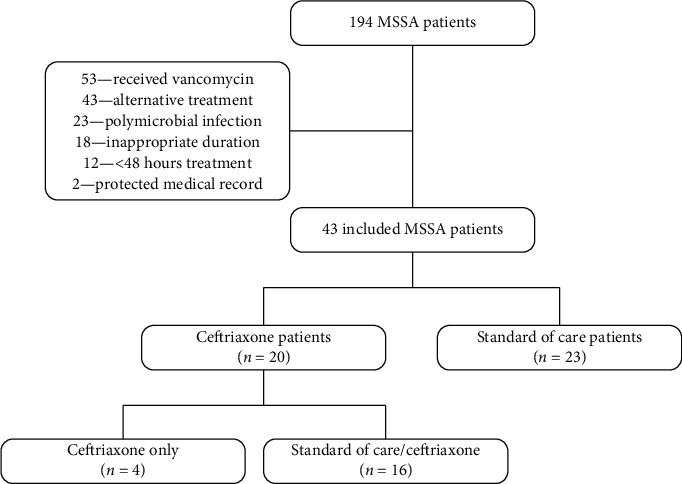
Flow diagram for study participants.

One hundred ninety-four patients were screened for inclusion with 43 (22.1%) patients meeting inclusion criteria (Figure 1). Twenty-three (53.5%) patients received SOCT only; 16 (37.2%) patients received SOCT before transitioning to ceftriaxone; 4 (9.3%) patients received only ceftriaxone. Due to the limited number of patients in the ceftriaxone arm, those patients were analyzed with patients who were transitioned to ceftriaxone after initial treatment with SOCT.

Baseline characteristics were similar between patients receiving SOCT and patients receiving ceftriaxone (Table 1) with a median age of 45 [36–56] years. Males represented 62.8% of the included patients. Close to half the patients had a community-acquired infection (48.8%), with the most common sources of infection being bone/joint (25.6%), central line catheter (20.9%), and skin/soft tissue or wound (16.3%) (Table 2). The source was able to be controlled in 76.7% of patients. The median duration of positive blood culture was 5 [3–6] days until clearance, with only 11.6% of patients having a recurrent bloodstream infection. Patients treated with nafcillin received 2 grams IV every 4 hours; cefazolin was given as 2 grams IV every 8 hours and adjusted for renal function. Ceftriaxone was given as 2 grams IV every 24 hours. Patients who transitioned from SOCT to ceftriaxone received SOCT for a median of 4.5 [0–9 IQR] days prior to switching therapy. Only 3 patients were initiated on ceftriaxone prior to blood culture clearance. The infectious diseases team was consulted in 37 (86%) patients. Twelve (27.9%) patients required admission to the intensive care unit but stayed for a median of less than 24 hours. Five (11.6%) of these patients required mechanical ventilation and vasopressor use; however, no patients expired within 7 days of first positive blood culture.

The primary composite outcome of clinical cure was achieved in 28 (65.1%) patients by day 7 of therapy (Table 3), with more patients achieving clinical cure in the SOCT group than the ceftriaxone group (78.3% vs 50%, *P*=0.052). WBC count normalization within 7 days occurred in significantly more patients in the SOCT group than in the ceftriaxone group (87% vs 60%, *P*=0.043); however, the time to WBC normalization was not significantly different between groups (3 [1–6] days SOCT vs 3.5 [1–12] days ceftriaxone, *P*=0.455). Thirty-seven (86%) patients achieved microbiological cure. The median length of hospital stay was 12 [8–23] days and was not significantly different between groups.

A majority of the patients were discharged home (67.4%) and were not readmitted or retreated for MSSA bacteremia within 30 days (76.7%) (Table 3). There were 5 (11.6%) patients who experienced a recurrent MSSA bacteremia within 60 days. The median time of follow-up post-discharge was 18 [13–24] days with patients in the SOCT group following up significantly sooner than the ceftriaxone group (12.5 [3.5–14] days vs 23 [18–47] days, *P*=0.003).

## 4. Discussion

In this study, we evaluated SOCT as compared to ceftriaxone or SOCT followed by ceftriaxone. Clinical cure, a composite endpoint, was not statistically different between groups. Although there was an almost 30% difference numerically, this was largely driven by white blood count resolution in 7 days, which was significantly different between groups. The 10 patients who did not meet the day 7 endpoint had a mean white blood count normalization within 14 days. The other two components of the composite endpoint (cultures cleared in 7 days and temperature normalization in 7 days) were not different between groups. It is important to note that, while cultures cleared in both groups, patients in both groups received a median of 5 days of SOCT, which is likely responsible for clearing the blood cultures. Mortality and readmissions were not different between groups. Interestingly, both hospital length of stay and MSSA bacteremia-related length of stay were numerically longer in the ceftriaxone group, but this was not statistically significant. These results suggest that, after cultures are clear, ceftriaxone may be appropriate follow-up or continuation therapy for patients initially hospitalized with MSSA bacteremia.

Few studies have evaluated ceftriaxone versus SOCT for MSSA bloodstream infections, and all have small sample sizes [17, 22]. Despite this, we found comparable microbiological cure rates for both treatment arms (SOCT [91.3% vs 94.1%] and ceftriaxone [80% vs 95.2%]) and no differences between the groups in clinical cure rates [22]. However, Patel and colleagues' study (*n* = 93) was performed at a VA Hospital and included a predominately older all-male population (median age = 68 years SOCT, 63 years ceftriaxone), whereas our study had an evenly distributed gender as well as a much younger population (median age = 45 years). Additionally, the majority of their patients received ceftriaxone in an ambulatory setting, whereas all of our patients were treated initially as inpatients, regardless of treatment group. Another evaluation of ceftriaxone for MSSA bloodstream infections (*n* = 71) performed by Carr and colleagues also took place at a VA Medical Center and found similar failure rates [17]. However, in this study, failure was associated with ceftriaxone usage despite similar durations of bacteremia and days of empiric therapy to our study. Lastly, a study by Paul and colleagues evaluated outcomes for beta-lactams against MSSA bacteremia which determined that odds of death were lower for oxacillin or cefazolin versus other beta-lactams [23]. However, data were not provided on patient characteristics or specific outcomes with ceftriaxone treated patients. If we were to remove WBC from the definition of clinical cure, our clinical cure rates are more comparable at 80% for ceftriaxone treated patients and 78% for SOCT patients.

This study is not without limitations. First, there were a limited number of patients who qualified for inclusion. This is because patients who received vancomycin or other agents with MSSA coverage (piperacillin-tazobactam, cefepime, etc.), which are routinely used at our hospital, were excluded. Second, only four patients received ceftriaxone as initial therapy. As a result, conclusions cannot be derived from this information regarding ceftriaxone as initial therapy. In addition, both groups received SOCT initially, further limiting the ability to determine the clinical utility of ceftriaxone before cultures have cleared. Next, a composite endpoint was used to define “clinical cure,” which may have impacted significance and may not correlate with markers used to determine cure or resolution in clinical practice. Finally, readmission rates, reoccurrences, and mortality were determined from our hospital system. Patients who presented to outside hospitals or were treated as outpatients may not be appropriately represented in these data.

In summary, there was no difference in clinical cure rates between SOCT and ceftriaxone for MSSA bacteremia. Ceftriaxone poses a reasonable alternative to consider for MSSA bacteremia when cost and feasibility are concerns for outpatient parenteral therapy after initial clearance of bloodstream infections.

## Figures and Tables

**Table 1:** Baseline demographics.

Variable presented as #(%) or median (IQR)	Ceftriaxone (*n* = 20)	SOCT (*n* = 23)	*P* value
Age	43.5 [35.25–57.5]	45 [36–55]	0.715
Sex, male	10 (50)	17 (73.9)	0.106
Race			
White	9 (45)	9 (39.1)	0.697
Black	11 (55)	14 (60.9)	0.697
Previous hospital stay within 6 months	7 (35)	7 (30.4)	0.750
Co-morbidities			
Diabetes mellitus	10 (50)	9 (39.1)	0.474
Hypertension	9 (45)	15 (65.2)	0.183
Congestive heart failure	3 (15)	5 (21.7)	0.704
COPD	0 (0)	1 (4.3)	1.000
Chronic kidney disease	2 (10)	8 (34.8)	0.076
Persons who inject drugs	2 (10)	5 (21.7)	0.420
Immunocompromised	3 (15)	5 (21.7)	0.704
Major surgical procedure	7 (35)	2 (8.7)	0.059

COPD = chronic obstructive pulmonary disease.

**Table 2:** Infection characteristics.

Variable presented as #(%) or median (IQR)	Ceftriaxone (*n* = 20)	SOCT alone (*n* = 23)	*P* value
Infection acquisition			
Community	11 (55)	10 (43.5)	0.451
Healthcare	5 (25)	9 (39.1)	0.324
Nosocomial	4 (20)	4 (17.4)	1.000
Infection source			
Bone/joint	4 (20)	7 (30.4)	0.434
Central line	3 (15)	6 (26.1)	0.467
Central nervous system	1 (5)	0 (0)	0.465
Infective endocarditis (native valve)	0 (0)	1 (4.3)	1.000
Respiratory tract	0 (0)	3 (13)	0.236
Skin/soft tissue/wound	3 (15)	4 (17.4)	1.000
Surgical site	2 (10)	0 (0)	0.210
Unknown	3 (15)	1 (4.3)	0.323
Source control			
Controlled	15 (75)	18 (78.3)	1.000
Not controlled	3 (15)	3 (13)	1.000
Unknown	2 (10)	2 (8.7)	1.000
Time to bloodstream clearance, days	5 [3–6]	4 [4–6]	0.892
Infectious diseases consult	19 (95)	18 (78.3)	0.192

**Table 3:** Clinical outcomes.

Variable presented as #(%) or median (IQR)	Ceftriaxone (*n* = 20)	SOCT alone (*n* = 23)	*P* value
Clinical cure	10 (50)	18 (78.3)	0.052
WBC normal in 7 days	12 (60)	20 (87)	0.043
Temp normal in 7 days	20 (100)	23 (100)	1.000
Culture clear in 7 days	16 (80)	18 (78.3)	1.000
Microbiological cure	16 (80)	21 (91.3)	0.393
Recurrence within 60 days	2 (10)	3 (13)	1.000
Hospital length of stay	16.5 [9–22.75]	12 [8–26]	0.669
MSSA length of stay	16 [8.25–22.75]	11 [8–19]	0.479
Discharge disposition			
Death	1 (5)	1 (4.3)	1.000
Home	13 (65)	16 (69.6)	0.750
SNF/Rehab	6 (30)	6 (26.1)	0.775
Alive, not readmitted or retreated	16 (80)	17 (73.9)	0.728
Infection-related readmission	2 (10)	4 (17.4)	0.669
Not infection-related readmission	2 (10)	1 (4.3)	0.590

WBC = white blood cell; MSSA = methicillin-susceptible *Staphylococcus aureus*; SNF = skilled nursing facility; Rehab = rehabilitation facility.

## Data Availability

No data were used to support this study.
